# A peptide from *Porphyra yezoensis* stimulates the proliferation of IEC-6 cells by activating the insulin-like growth factor I receptor signaling pathway

**DOI:** 10.3892/ijmm.2014.2037

**Published:** 2014-12-11

**Authors:** MIN-KYEONG LEE, IN-HYE KIM, YOUN-HEE CHOI, TAEK-JEONG NAM

**Affiliations:** 1 Department of Food Science and Nutrition, Pukyong National University, Busan 608-737, Republic of Korea; 2 Institute of Fisheries Science, Pukyong National University, Busan 619-911, Republic of Korea

**Keywords:** *Porphyra yezoensis*, proliferation, insulin-like growth factor-I receptor signaling pathway

## Abstract

*Porphyra yezoensis* (*P. yezoensis*) is the most noteworthy red alga and is mainly consumed in China, Japan and Korea. In the present study, the effects of a *P. yezoensis* peptide (PY-PE) on cell proliferation and the associated signaling pathways were examined in IEC-6 rat intestinal epithelial cells. First, the MTS assay showed that PY-PE induced cell proliferation in a dose-dependent manner. Subsequently, the mechanism behind the proliferative activity induced by PY-PE was determined. The insulin-like growth factor-I receptor (IGF-IR) signaling pathway was the main focus as it plays an important role in the regulation of cell growth and proliferation. PY-PE increased the protein and mRNA expression of IGF-IR, insulin receptor substrate-1, Shc and PY-99. In addition, PY-PE stimulated extracellular signal-regulated kinase phosphorylation and phosphatidylinositol 3-kinase/Akt activation but inhibited p38 and c-Jun N-terminal kinase phosphorylation. Furthermore, PY-PE treatment increased protein and mRNA expression levels of activator protein-1, which regulates cell proliferation and survival, in the nuclear fraction. These results have significant implications for understanding the role of cell proliferation signaling pathways in intestinal epithelial cells.

## Introduction

Red seaweeds have attracted increasing attention in recent years through research aimed to develop new medicines and healthy diets from bioactive compounds (1). *Porphyra yezoensis* (*P. yezoensis*) is a critical alga that is mainly consumed in Korea, China and Japan. This alga is an important source of physiologically active substances, such as sulfated polysaccharides, polyphenols and peptides, with biological effects including antitumor (2), anti-inflammatory (3), antioxidant (4) and blood pressure effects (5). The majority of studies on the biological activities of *P. yezoensis* have been conducted *in vivo*. In the present study, the induction of proliferation was examined in intestinal epithelial cells by a peptide from *P. yezoensis*.

Activation of the insulin-like growth factor-I receptor (IGF-IR) via autocrine, paracrine and endocrine mechanisms appears to play an important role in regulating cell growth, proliferation and differentiation (6). The insulin receptor substrate (IRS) family (IRS-1 to IRS-4) and Shc are the best characterized substrates of IGF-IR. The IRS proteins are docking proteins that potentially bind to IGF-IR and recruit several effector proteins through Src homology 2 (SH2) domain interactions (7,8). Shc is a substrate of tyrosine kinase receptors, non-receptor kinases and certain phosphatases (9,10). Upon stimulation with IGF-I, tyrosine phosphorylation sites in the IRS proteins bind to phosphotyrosine-containing binding motifs (YXXM) within SH2 domains in several downstream signaling molecules, including growth factor receptor-bound protein 2, SH2-containing protein-tyrosine phosphatase 2 and the p85 regulatory subunit of phosphatidylinositol 3-kinase (PI3K) (11). Receptor activation leads to activation of various signaling pathways, including mitogen-activated protein kinase (MAPK) cascades (12). PI3K is a critical regulatory protein involved in intracellular signal transduction processes and controlling major cellular functions (13). Activated PI3K catalyzes the phosphorylation of the membrane phospholipid phosphatidylinositol 4,5-bisphosphate to generate phosphatidylinositol 3,4,5-trisphosphate, thereby producing a lipid-binding site on the cell membrane for the serine/threonine kinase Akt (14). Akt is activated by phosphorylation at Thr^308^ and Ser^473^ residues by two phosphoinositide-dependent protein kinases, PDK1 and PDK2 (15). Akt controls cell survival, proliferation, growth and motility (16). In mammalian cells, MAPK cascades constitute a large kinase network that regulates a variety of biological processes, including cell growth, proliferation, differentiation and inflammatory responses (17). The p42/p44 MAPK [extracellular signal-regulated kinase (ERK)] signal transduction pathway is activated by various mitogens. By contrast, c-Jun N-terminal kinase (JNK) and p38 pathways are mainly activated by cellular stress and inflammatory cytokines. Notably, ERK is associated with cell proliferation and growth (18,19).

Activator protein-1 (AP-1) comprises homodimers and heterodimers composed of basic-region leucine zipper proteins that belong to the Jun (c-Jun, v-Jun and Jun D) and Fos (c-Fos, v-Fos and Fos B) families, as well as the associated activating transcription factors (ATF2, B-ATF and ATF3/LRF1) (20,21). AP-1 regulation is induced by various stimuli, including growth factors, cytokines and ultraviolet (UV) irradiation. In addition, three different types of MAPKs (ERK, JNK and fos-regulating kinase) contribute to induction of AP-1 activity in response to a diverse array of extracellular stimuli (22). Jun-Fos (heterodimeric) and Jun-Jun (homodimeric) complexes preferentially bind to the 12-*O*-tetredecanoylphorbol-13-acetate-responsive element (23). The AP-1 complex mediates responses to cellular signals by binding to DNA and inducing gene transcription changes leading to physiological activity in the cell. AP-1 thereby regulates numerous cellular processes, including cell proliferation, differentiation and stress responses (24).

In the present study, a peptide from *P. yezoensis* (known as PY-PE) is shown to have proliferative effects on IEC-6 intestinal epithelial cells. The intracellular mechanism of PY-PE was determined, focusing on the IGF-IR signaling pathway, which is involved in the regulation of cellular proliferation and differentiation.

## Materials and methods

### Preparation of PY-PE

**Figure 1:**
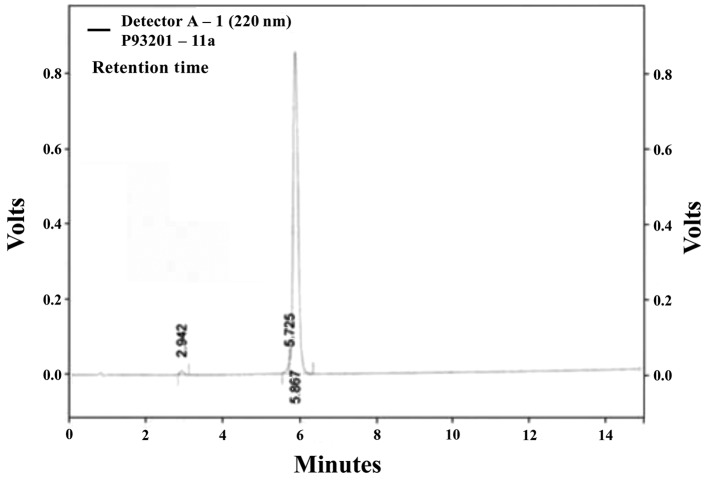
Purification of the peptide from *P. yezoensis* (PY-PE) by Shiseido Capcell Pak C18 column chromatography.

PY-PE (A-L-E-G-G-K-S-S-G-G-G-E-A-T-R-D-P-E-P-T) was synthesized by Peptron, Inc. (Daejeon, Korea). Purification of PY-PE was performed using the Shimadzu Prominence HPLC apparatus and a C18 column (Capcell Pak; Shiseido, Tokyo, Japan) in 0.1% trifluoroacetic acid (TFA)/water and a gradient of 10–70% acetonitrile (0% acetonitrile in 2 min, 0–30% acetonitrile in 10 min, 30–90% acetonitrile in 2 min) in 0.1% TFA, with a flow rate of 1 ml/min and UV detection at 220 nm, controlled using the software package Class-VP, 6.14 (Kyoto, Japan). The molecular weight of PY-PE was determined to be 1,916 kDa (Fig. 1) using a mass spectrometer (HP 1100 Series LC/MSD; Agilent Technologies, Santa Clara, CA, USA).

### Cell culture

IEC-6 rat small intestinal epithelial cells (ATCC CRL-1592) were obtained from the American Type Culture Collection (Rockville, MD, USA). Cells were maintained in a humidified 5% CO_2_ incubator at 37°C in Dulbecco’s modified Eagle’s medium supplemented with 10% fetal bovine serum (HyClone, Inc., South Logan, UT, USA), 100 U/ml penicillin and 100 mg/ml streptomycin. The medium was replaced every 2 days.

### Cell proliferation assay

Cell proliferation was estimated using a CellTiter 96^®^ aqueous non-radioactive cell proliferation assay (Promega, Madison, WI, USA), which is based on the cleavage of 3-(4,5-dimethylthiazol-2-yl)-5-(3-carboxymethoxyphenyl)-2-(4-sulfonyl)-2H-tetrazolium (MTS) into a formazan product that is soluble in tissue culture medium. Cells were seeded in 96-well plates at 1×10^4^ cells/well in 100 *μ*l medium and attached for 24 h. Attached cells were maintained in serum-free medium (SFM) for 4 h and were subsequently treated with PY-PE (125–1,000 ng/ml) for 24 h. The cells were incubated with 10 *μ*l MTS solution for 30 min, and the absorbance of each well was measured at 490 nm using a SpectraMax 340PC microplate reader (Molecular Devices, Sunnyvale, CA, USA).

### Extraction of whole-cell protein lysates

IEC-6 cells were plated in 100-mm dishes at a density of 2×10^4^ cells/ml and cultured to 60% confluence at 37°C. The cells were subsequently incubated for 24 h in SFM containing 0, 125, 250, 500 or 1,000 ng/ml PY-PE. Cells were washed with phosphate-buffered saline and suspended on ice in lysis buffer [50 mM Tris, 5 mM EDTA, 150 mM NaCl and 1% Triton X-100 (pH 7.5)] containing protease inhibitors (1 mg/ml aprotinin, 1 mg/ml leupeptin, 1 mg/ml pepstatin A, 200 mM Na_3_VO_4_, 500 mM NaF and 100 mM PMSF). The extracts were centrifuged at 14,000 rpm (10,770 × g) for 10 min, and the supernatant was used for western blot analysis.

### Extraction of nuclear lysates

Cells were treated and harvested as described above, lysed with hypotonic lysis buffer [10 mM 4-(2-hydroxyethyl)-1-piperazineethanesulfonic acid)(HEPES) (pH 7.9), 10 mM KCl and 1.5 mM MgCl_2_], and incubated for 15 min at 4°C. Cells were lysed further by the addition of 2.5% NP-40 and incubated for 10 min at 4°C. After 10 min, nuclei were collected by centrifugation at 5,000 rpm (1,593 × g) for 5 min at 4°C. Nuclear proteins were resuspended in extraction buffer [10 mM HEPES (pH 7.9), 100 mM NaCl, 1.5 mM MgCl_2_, 0.1 mM EDTA and 0.1 mM dithiothreitol] and incubated for 20 min at 4°C. The extracts were centrifuged at 14,000 rpm (10,770 × g) for 10 min and the supernatant was used for western blot analysis.

### Western blot analysis

Protein extracts (30 *μ*g) were separated by 7.5–12.5% sodium dodecyl sulfate-polyacrylamide gel electrophoresis and transferred to polyvinylidene fluoride membranes (Millipore, Billerica, MA, USA). Membranes were blocked with 1% bovine serum albumin (BSA) in TBS-T [10 mM Tris-HCl, 150 mM NaCl (pH 7.5) and 0.1% Tween-20] and incubated overnight with the indicated primary antibodies (diluted 1:500, 1:1,000 or 1:2,000; Santa Cruz Biotechnology, Inc., Santa Cruz, CA, USA) in TBS-T containing 1% BSA with gentle agitation at 4°C. The secondary antibody was a horseradish peroxidase-conjugated goat anti-mouse or anti-rabbit antibody (A90-116P; diluted 1:10,000; Bethyl Laboratories, Inc., Montgomery, TX, USA). The signals were detected using an enhanced chemiluminescence Western blotting kit (Thermo Fisher Scientific, Inc., Rockford, IL, USA).

### Reverse transcription-polymerase chain reaction (RT-PCR)

The mRNA expression levels of specific genes were evaluated by RT-PCR. IEC-6 cells were seeded in 100-mm dishes at a density of 2×10^4^ cells/well and were cultured for 24 h, after which the medium was replaced with SFM containing PY-PE (125, 250, 500 or 1,000 ng/ml) for 24 h. RNA was extracted from cells using the TRIzol reagent (Invitrogen Life Technologies, Carlsbad, CA, USA) and quantified using oligo(dT) primers (Intron Biotechnology Co., Ltd., Seongnam, Korea). cDNA was reverse transcribed from the RNA and subjected to amplification using a PCR kit (dNTP mix, 10X Ex Taq Buffer and Ex Taq; Takara Bio, Inc., Shiga, Japan) with primers (Table I) in 0.1% diethylpyrocarbonate water. PCR products were resolved on 1% agarose gels. Gels were stained with 10 mg/ml ethidium bromide to visualize the amplification products.

### Statistical analysis

Significant differences among multiple mean values were assessed by analysis of variance using SPSS version 10.0 (SPSS Inc., Chicago, IL, USA). P<0.05 was considered to indicate a statistically significant difference.

## Results

### Proliferative effect of PY-PE in IEC-6 cells

**Figure 2:**
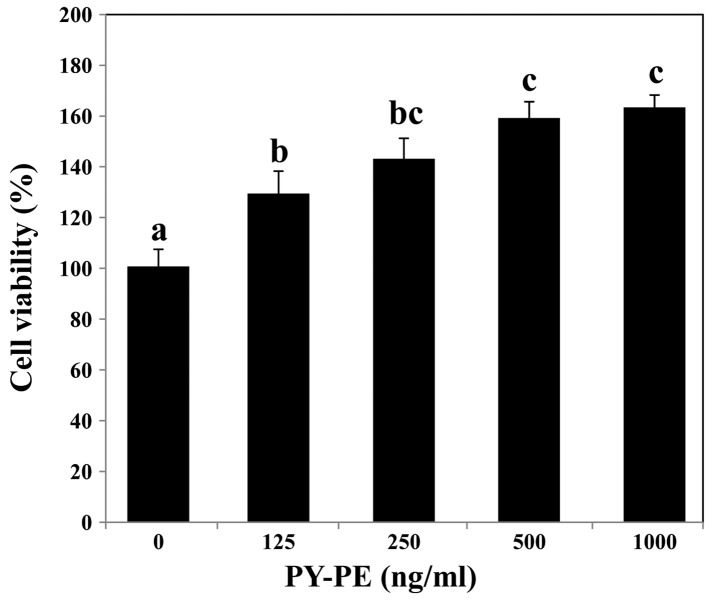
Proliferative effect of *P. yezoensis* (PY-PE) on IEC-6 cells. Cells were seeded in 96-well plates at a density of 1×10^4^ cells/well with 10% fetal bovine serum-supplemented medium. After incubation for 24 h, the cells were serum-starved for 4 h and treated with PY-PE at the indicated concentrations for 24 h. The results are presented as the means ± standard deviation of the three independent experiments.

Through mass spectrometry, a 1,916-kDa compound was identified from *P. yezoensis*, which was designated as PY-PE (Fig. 1). The proliferative effect of PY-PE on IEC-6 cells was confirmed using MTS assay. Treatment with PY-PE for 24 h increased cell viability in a dose-dependent manner (Fig. 2).

### Effect of PY-PE treatment on the expression of IGF-IR-related proteins

**Figure 3:**
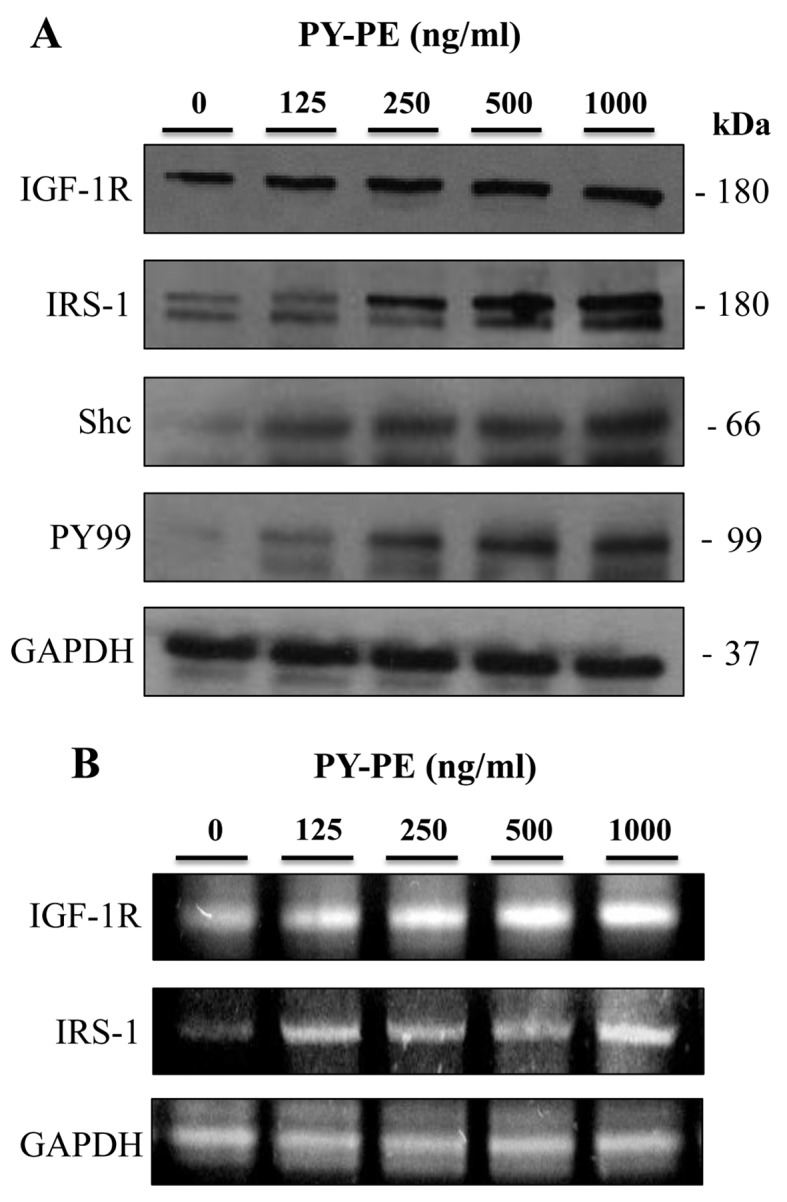
Effect of *P. yezoensis* (PY-PE) treatment on IGF-IR, IRS-1, Shc and PY-99 protein and mRNA expression levels in IEC-6 cells. Protein expression was examined by western blotting, and cDNA was subjected to RT-PCR analysis. (A) Protein expression levels were increased upon incubation with PY-PE for 24 h. (B) mRNA expression levels were also increased. IGF-IR, insulin-like growth factor-I receptor; IRS-1, insulin receptor substrate-1; RT-PCR, reverse transcription-polymerase chain reaction..

To confirm the mechanism of PY-PE-induced proliferation in IEC-6 cells, the effects of PY-PE were examined on IGF-IR signaling pathway-related proteins. The protein and mRNA expression levels of IGF-IR, IRS-1, Shc and PY-99 in IEC-6 cells treated with PY-PE (125, 250, 500 and 1,000 ng/ml) for 24 h were determined by western blotting and RT-PCR. Treatment with PY-PE dose-dependently upregulated the protein (Fig. 3A) and mRNA (Fig. 3B) expression levels of IGF-IR, IRS-1, Shc and PY-99. IGF-IR stimulates the proliferation of various cell types and inhibits apoptosis (25).

### Effect of PY-PE treatment on the expression of MAPK signaling pathway proteins

**Figure 4:**
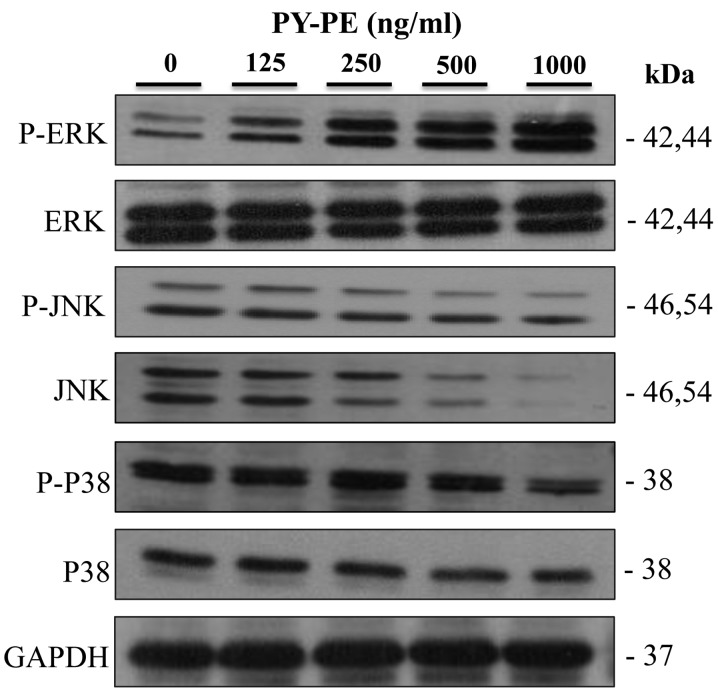
Effect of *P. yezoensis* (PY-PE) treatment on the mitogen-activated protein kinase (MAPK) signaling pathway. Whole-cell extracts were prepared and analyzed by western blotting using anti-phospho-extracellular signal-regulated kinase 1/2 (ERK1/2), anti-ERK, anti-phospho-c-Jun N-terminal kinase (JNK), anti-JNK, anti-phospho-p38, anti-p38 and anti-glyceraldehyde 3-phosphate dehydrogenase (GAPDH) antibodies.

To further investigate the downstream signals regulated by IGF-IR activation, the expression levels of the MAPK family proteins (ERK1/2, JNK and P38) in IEC-6 cells treated with PY-PE (125, 250, 500 and 1,000 ng/ml) for 24 h were determined by western blot analysis. Treatment with PY-PE dose-dependently increased the protein expression level of ERK1/2. By contrast, PY-PE treatment inhibited the activation of JNK and p38 in dose-dependent manners (Fig. 4). These results suggest that ERK1/2 plays an important role in the proliferation of IEC-6 cells.

### Effect of PY-PE treatment on the expression of PI3K-Akt signaling pathway proteins

**Figure 5:**
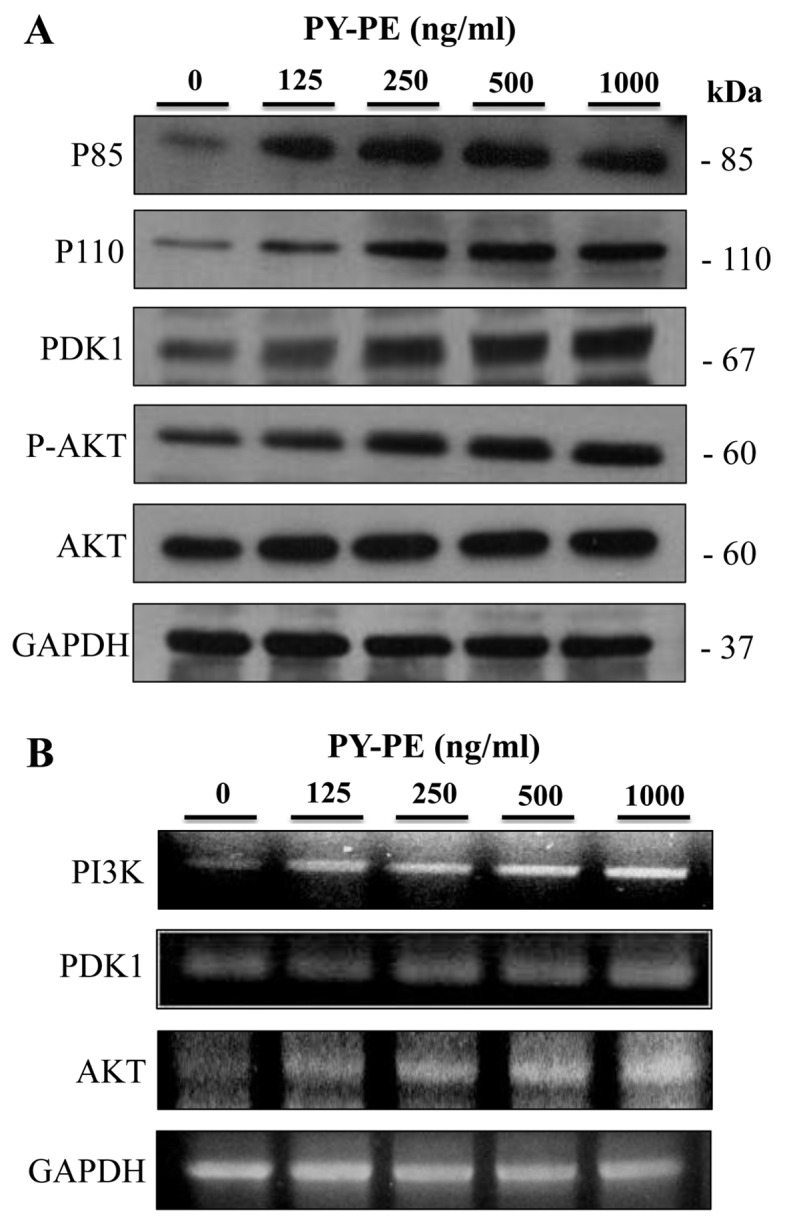
Effect of *P. yezoensis* (PY-PE) treatment on phosphatidylinositol 3-kinase (PI3K) (p85 and p110), Akt and phosphoinositide-dependent protein kinase 1 (PDK1) (A) protein and (B) mRNA expression levels in IEC-6 cells. Whole-cell extracts were prepared and analyzed by western blot analysis using anti-p85, anti-p110, anti-PDK1, anti-phospho-Akt, anti-Akt and anti-glyceraldehyde 3-phosphate dehydrogenase (GAPDH) antibodies.

The expression levels of the PI3K-Akt signaling pathway intermediates were examined by western blotting and RT-PCR. PY-PE treatment for 24 h resulted in increased protein and mRNA expression levels of p85, p110, PDK1 and p-Akt compared to the controls (Fig. 5).

### Effect of PY-PE treatment on the expression of AP-1

**Figure 6:**
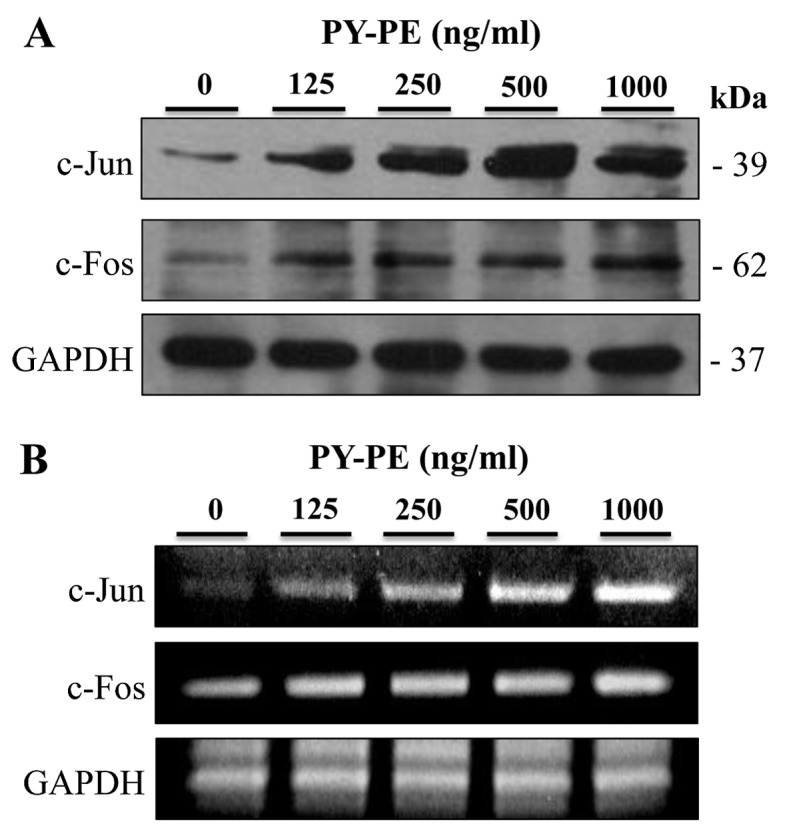
Effect of *P. yezoensis* (PY-PE) treatment on c-Jun and c-Fos (A) protein and (B) mRNA expression levels in IEC-6 cells. Cells were treated with PY-PE after preincubation with serum-free medium (SFM) for 4 h. Nuclear extracts were prepared and analyzed by western blotting using anti-c-Jun, anti-c-Fos and anti-glyceraldehyde 3-phosphate dehydrogenase (GAPDH) antibodies.

IGF-IR activates the PI3K/Akt and p42/p44 MAPK pathways, which regulate the activation of transcription factors, such as AP-1 (26). AP-1 regulates cell proliferation and survival. Therefore, AP-1 protein and mRNA expression levels were examined by western blot analysis and RT-PCR, respectively. PY-PE treatment of IEC-6 cells upregulated the protein and mRNA expression levels of c-Jun and c-fos compared to the controls (Fig. 6).

## Discussion

Numerous types of seaweed are important sources of physiologically active substances, such as peptides and polysaccharides, and have shown medicinal benefits, including anti-ulcer, antitumor, antibacterial, antioxidant and antiviral activities (27–29).

The purpose of the present study was to determine whether PY-PE induced proliferation of IEC-6 cells and to identify the associated signals. In an MTS assay, exposure to PY-PE for 24 h had dose-dependent effects on the proliferation of IEC-6 cells (Fig. 2). The mechanism associated with this proliferative effect was subsequently examined. The regulation of cell proliferation is dependent on various signaling pathways. In the present study, the IGF-IR signaling pathway was the main focus.

IGF-IR regulates the biological activity of IGF-I and plays critical roles in cell growth, differentiation and proliferation. Additionally, adaptor proteins, such as IRS-1, Shc and PY-99, have all been indicated in sending signals to the cell nucleus (7,30). The results of the present study showed that PY-PE increased the protein and mRNA expression levels of IGF-IR, IRS-1, Shc and PY-99 (Fig. 3). Following IGF-IR activation, downstream signaling pathways, including the PI3K and p42/p44 MAPK pathways, are activated (31). The MAPK family in mammalian cells includes ERK-1/2, JNK, SAPK and p38 kinase (32). ERK1/2 is activated primarily in response to mitogens and growth factors. This pathway plays an important role in cell proliferation, growth and survival (33–35). By contrast, JNK and p38 are mostly activated by cellular stress and by cytokines (36). In the present study, in accordance with PY-PE-induced cell proliferation, ERK1/2, a critical mediator that regulates cell growth and proliferation, was activated by exposure to PY-PE. By contrast, PY-PE treatment did not induce phosphorylation of JNK or p38 (Fig. 4).

The present results showed that IGF-IR activation also contributes to PY-PE-induced cell proliferation through activation of the PI3K/Akt pathway, a key intracellular signaling pathway that regulates multiple cellular processes, including cell survival, growth and proliferation (37). PI3K is a heterodimeric protein composed of two subunits: A p85 regulatory subunit (85 kDa) and a p110 catalytic subunit (110 kDa) (38,39). Once the p85 regulatory subunit is positioned appropriately, the p110 catalytic subunit of PI3K generates phosphatidylinositol 3,4,5-triphosphate, which in turn activates various downstream targets, including the serine/threonine kinase Akt (40,41). In the present study, PY-PE increased the protein and mRNA expression levels of p85, p110 and PDK1, as well as Akt phosphorylation (Fig. 5).

In a wide range of systems, it has been well established that growth factor ligation leads to activation of the p42/p44 MAPK and PI3K/Akt pathways, resulting in the activation of various transcription factors (42). For instance, platelet-derived growth factor activates the p42/p44 MAPK pathway, which modulates AP-1 activation, in NIH 3T3 mouse fibroblasts (43,44). Therefore, PY-PE-induced cell proliferation was assumed to involve AP-1. These results show that PY-PE increased the protein and mRNA expression levels of c-Jun and c-fos (Fig. 6).

In conclusion, PY-PE stimulated the proliferation of IEC-6 normal intestinal epithelial cells. This effect was also associated with the IGF-IR signaling pathway. Therefore, PY-PE may be a potential component of bio-functional foods with a proliferative effect on intestinal epithelial cells.

## Figures and Tables

**Table I:** Oligonucleotide sequences of the primers used in RT-PCR.

Gene name	Primer sequence (5′-3′)
*IGF-IR*	F: AAA-TGT-GCC-CGA-GCG-TGT-G R: TGC-CCT-TGA-AGA-TGG-TGC-ATC
*IRS-1*	F: ACT-TGA-GCT-ATG-ACA-CGG-CT R: GGT-TGG-AGC-AAC-TGG-ATG-AA
*PI3K*	F: GCC-GAA-CAC-CTT-TTT-GAG-TC R: AGG-AGC-GGT-ACA-GCA-AAG-AA
*PDK1*	F: AAG-GGT-ACG-GGC-CTC-TCA-AA R: CCC-ACG-TGA-TGG-ACT-GAA-AGA
*Akt*	F: CAA-CTT-CTC-TGR-GGC-GCA-GTG R: GAC-AGG-TGG-AAG-AAC-AGC-TCG
*c-Jun*	F: TCA-AAA-TGT-TTG-CAA-CTG-CTG-CG R: ATG-ACT-GCA-AAG-ATG-GAA-ACG
*c-Fos*	F: GGA-GAA-TCC-GAA-GGA-AGG R: GCT-TGG-GCT-CAG-GGT-CAT-TG
*GAPDH*	F: CAG-CCG-AGC-CAC-ATC-G R: TGA-GGC-TGT-TGT-CAT-ACT-TCT-C

F, forward; R, reverse; RT-PCR, reverse transcription-polymerase chain reaction; IGF-IR, insulin-like growth factor-I receptor; IRS-1, insulin receptor substrate-1; PI3K, phosphatidylinositol 3-kinase; PDK1, phosphoinositide-dependent protein kinase 1; GAPDH, glyceraldehyde 3-phosphate dehydrogenase.
